# Molecular Mechanisms Underlying the Relationship between Obesity and Male Infertility

**DOI:** 10.3390/metabo11120840

**Published:** 2021-12-04

**Authors:** Federica Barbagallo, Rosita A. Condorelli, Laura M. Mongioì, Rossella Cannarella, Laura Cimino, Maria Cristina Magagnini, Andrea Crafa, Sandro La Vignera, Aldo E. Calogero

**Affiliations:** Department of Clinical and Experimental Medicine, University of Catania, 95131 Catania, Italy; federica.barbagallo11@gmail.com (F.B.); rosita.condorelli@unict.it (R.A.C.); lauramongioi@hotmail.it (L.M.M.); rossella.cannarella@phd.unict.it (R.C.); lauracimino@hotmail.it (L.C.); crymaga@hotmail.it (M.C.M.); crafa.andrea@outlook.it (A.C.); acaloger@unict.it (A.E.C.)

**Keywords:** obesity, male fertility, sperm parameters, adipokines, sirtuins

## Abstract

In recent decades, the worldwide prevalence of obesity has risen dramatically and is currently estimated to be around 20%. Obesity is linked to an increased risk of comorbidities and premature mortality. Several studies have shown that obesity negatively impacts male fertility through various mechanisms. This review aims to investigate the molecular mechanisms through which obesity impairs male reproduction, including obesity-associated hypogonadism and its effects on spermatogenesis, chronic inflammation, and oxidative stress. Obesity negatively impacts both conventional and biofunctional sperm parameters, and it also induces epigenetic changes that can be transferred to offspring. Moreover, obesity-related diseases are linked to a dysregulation of adipocyte function and micro-environmental inflammatory processes. The dysregulated adipokines significantly influence insulin signaling, and they may also have a detrimental effect on testicular function. Sirtuins can also play an important role in inflammatory and metabolic responses in obese patients. Understanding the molecular mechanisms that are involved in obesity-induced male infertility could increase our ability to identify novel targets for the prevention and treatment of obesity and its related consequences.

## 1. Introduction

Obesity is defined as an abnormal or excessive accumulation of fat. According to the World Health Organization (WHO), a body mass index (BMI) that is greater than or equal to 25 kg/m^2^ is classified as overweight, a BMI greater than 30 kg/m^2^ is considered obesity, and a BMI greater than 40 kg/m^2^ is considered severe obesity [1].

In recent decades, the worldwide prevalence of obesity has risen dramatically, and nowadays, it is considered a global health issue [2]. The WHO has estimated that about 1.9 billion adults worldwide were overweight in 2016 and that around 650 million were obese. Obesity is linked to an elevated premature mortality risk [3] and an increased risk of comorbidities. These include cerebral–cardiovascular diseases, type 2 diabetes mellitus, hypertension, asthma, sleep apnea syndrome, psychiatric diseases, polycystic ovary syndrome (PCOS), nonalcoholic fatty liver disease (NAFLD), gastrointestinal reflux disease, gallbladder disease, osteoarthritis, and many other endocrine system disorders. All of these can negatively impact a person’s life expectancy and quality of life and may influence one’s reproductive and sexual health [4]. Obesity is also a risk factor for some neoplasias and has been associated with a poorer response to cancer therapy. Furthermore, it has a detrimental effect on severe acute respiratory syndrome coronavirus 2 (SARS-CoV-2) infection and the severity of the coronavirus disease (COVID-19) [5]. Therefore, obesity represents one of the most life-threatening, chronic health problems, as recognized by WHO.

Several studies have shown that obesity interferes with male reproductive function [6] through multiple mechanisms that are still not fully understood. Therefore, this review aims to investigate the relationship between obesity and male infertility, focusing on the molecular mechanisms through which it negatively affects spermatogenesis, sperm quality, and male reproduction.

## 2. Epidemiology of Male Fertility in Obesity

Previous population-based studies have reported an increased prevalence of abnormal sperm parameters in overweight and obese men [7,8,9]. In their meta-analysis including 115, 158 total participants, Campbell and colleagues reported that couples with an obese male partner have a significantly higher risk of infertility than couples with normal-weight male partners (OR = 1.66; 95% CI 1.53–1.79) [8]. Moreover, male obesity negatively affects the success of assisted reproductive technology (ART) [8]. In 2018, Mushtaq and colleagues reported that an increased BMI in men was associated with a significant reduction in pregnancy (OR 0.78, 95% CI 0.63 to 0.98, *p* = 0.03) and live birth (OR 0.88, 95% CI 0.82 to 0.95, *p* = 0.001) rates in intracytoplasmic sperm injection (ICSI) cycles [10]. Currently, there is increasing evidence that male obesity may be equally involved in the pathogenesis of infertility and embryo quality as female obesity is [11].

## 3. Hormonal Changes in Obesity

The testosterone (T) synthesis is controlled by the hypothalamic–pituitary–gonad (HPG) axis and is modulated by direct negative feedback from T. The pulsatile secretion of the gonadotropin-releasing hormone (GnRH) stimulates the release of the luteinizing hormone (LH) and the follicle-stimulating hormone (FSH). In the testis, LH acts on Leydig cells (LCs), stimulating T secretion, and FSH acts on Sertoli cells (SCs), inducing spermatogenesis [12].

Most obese males have impaired reproductive hormonal profiles compared to normal-weight men. The excessive visceral fat decreases the serum sex hormone-binding globulin (SHBG), total and free T, and inhibin B levels and increases the conversion of T into 17ß-estradiol (E_2_) due to greater aromatase activity [13].

Aromatase activity indeed increases with the body fat mass and further increases fat accumulation, creating a vicious cycle [12]. In obese male patients, the higher aromatase activity leads to an increased conversion of T into E_2_ and, in turn, decreases the T/E_2_ ratio [14]. Since estrogens are more biologically active than T, the increased levels of estrogens can inhibit the HPG axis, suppressing the activity of the kisspeptin neurons, further reducing T production [15]. Kisspeptin (encoded by the *KISS1* gene) plays a key role in the inhibitory effect of estrogens within the hypothalamus [16]. Estrogens inhibit LCs and SCs and, in turn, impair the secretion of T and spermatogenesis [12].

Furthermore, higher serum SHBG levels are one of the major causes of reduced T levels in obese male patients. SHBG is a glycoprotein that binds sex hormones, inhibiting their biological activity. In obese patients, hyperinsulinemia, which is induced by insulin resistance, may reduce the production of SHBG by the liver, resulting in estrogen demonstrating greater biological activity [17]. In addition, hyperinsulinemia has an inhibitory effect on spermatogenesis, increasing nuclear and mitochondrial DNA damage [18].

Obesity is also associated with lower levels of inhibin B. This is a growth-like factor that is produced by SCs that inhibits FSH secretion and that stimulates T secretion by LCs [11]. Inhibin B is a normal spermatogenesis marker. In obese male patients, the decreased levels of inhibin B indicate dysfunction of the seminiferous testicular compartment [19].

Lastly, adipose tissue stimulates the secretion of the pro-inflammatory cytokines and adipokines that inhibit T production. It has been reported that serum leptin is positively associated with BMI and adipose mass in healthy men, whereas it negatively correlates with serum T in overweight and obese subjects [20].

## 4. Obesity, Insulin Resistance, and Male Infertility

Insulin resistance plays a main role in obesity-induced hypogonadism. Insulin resistance is defined as the reduced sensitivity of cells to insulin effects in normal or elevated blood glucose levels. Consequently, the pancreas has to secrete higher amounts of insulin, and this results in a state of hyperinsulinemia. With the persistence of insulin resistance, hyperglycemia ensues and, in turn, causes glucose intolerance and ultimately type 2 DM (T2DM).

Visceral adipose tissue has been shown to strongly correlate with insulin resistance. Visceral obesity enhances the delivery of free fatty acids to the liver, leading to reduced hepatic insulin clearance. This further increases circulating insulin levels, leading to hyperinsulinemia. The increased production of adipokines and inflammatory mediators significantly influences insulin signaling in insulin-responsive tissues, promoting systemic insulin resistance and hyperinsulinemia. Cell culture studies have shown that insulin stimulates HPG axis activity both at central (hypothalamus and pituitary) and peripheral (testis) levels. Insulin resistance may also affect hypothalamic neurons and consequently GnRH secretion [21].

In obese patients, hyperinsulinemia that is induced by insulin resistance decreases SHBG secretion by the liver, resulting in greater biological estrogen activity [17]. Therefore, excess insulin may also have a detrimental effect on testicular function and androgenic status in obese adult men, giving rise to a “metabolic” form of male hypogonadism. In addition, hyperinsulinemia has an inhibitory effect on spermatogenesis, increasing nuclear and mitochondrial DNA damage [18]. However, a complex, bi-directional crosslink between visceral adipose tissue dysfunction, systemic insulin resistance, and testicular malfunctioning has been demonstrated. Indeed, hypogonadism levels further deteriorate insulin sensitivity and promote adipocyte proliferation and body fat increase [22]. Consequently, low testosterone levels can worsen obesity and promote the development of metabolic diseases in overweight and obese patients, creating a vicious circle [23].

## 5. Obesity and Conventional Sperm Parameters

Several previous studies have shown an alteration of sperm parameters in obese patients [13,24]. However, other studies reported controversial results [25,26,27]. Pauli and colleagues did not find any correlation between BMI and sperm concentration, seminal fluid volume, motility, or morphology [26]. Shayeb and colleagues described a decrease in the percentage of spermatozoa with normal morphology but not of sperm concentration and motility in obese men [27]. The discrepancy between the various studies could relate to several limitations in human studies, including confounding factors or differences in the populations that are selected by each study. In fact, lots of other lifestyle factors including smoking, the use of drugs or alcohol, or comorbidities can alter semen parameters. Moreover, studies that have been conducted with patients enrolled in fertility clinics may also have other confounding factors that are related to other reasons for infertility.

We have previously shown that overweight and to a greater extent obese men have abnormal conventional sperm parameters; in particular, asthenozoospermia and teratozoospermia [28]. A systematic review and meta-analysis performed by Sermondade et al. in 2013 that included 13,077 men showed that overweight and obesity are associated with a higher prevalence of azoospermia and oligozoospermia [24]. Chavarro and colleagues reported that a BMI > 25 kg/m^2^ correlates to a poorer total sperm count [13].

Several mechanisms have been hypothesized to explain obesity-induced sperm damage. The hormonal alterations that were described above certainly contribute to impairing spermatogenesis in obese patients. Intratubular T plays a key role in spermatogenesis since it is responsible for the adhesion of SCs to the developing germ cells [29]. In turn, SCs, the only somatic cells that are in direct contact with germ cells, provide the latter with nourishment and support. A reduction in intratubular androgens is also associated with the retention and phagocytosis of mature spermatids [29]. Inhibin B is an important marker of spermatogenesis. Indeed, its decreased levels are associated with seminiferous tubule dysfunction [26].

However, the dysregulation of sex hormones is not the only cause of infertility in obese males. A pivotal role is also played by heat-induced damage. Testicular thermal stress increases in obese men and is mainly due to fat accumulation in the suprapubic region and around the pampiniform plexus. The resulting increase in scrotal temperature reduces sperm concentration and motility and enhances oxidative stress and sperm DNA fragmentation (SDF) [30]. The role of oxidative stress and other molecular mechanisms that can contribute to the impairment of sperm parameters in obese patients will be discussed in the following paragraphs.

## 6. Obesity, Biofunctional Sperm Parameters, and Oxidative Stress

Although several studies have investigated the effect of obesity on conventional sperm parameters, few authors have explored the relationship between body weight and biofunctional sperm parameters. The evaluation of non-conventional sperm parameters can provide important information that could not be obtained through classical seminal analysis alone and that, in turn, may be very useful to identify the cause(s) that are responsible for the failure to conceive negative pregnancy outcomes.

SDF is the most studied biofunctional sperm parameter to ensure normal fertilization, embryo development, and implantation [31]. Abnormal SDF can be caused by extrinsic factors such as smoking and environmental pollutants [32,33,34] or intrinsic factors, including abortive apoptosis and oxidative stress [35]. Lifestyle and environmental factors cause an increase in SDF [35]. In a previous study, we showed that the biofunctional sperm parameters are altered in overweight/obese men. In particular, obese patients showed an increased percentage of spermatozoa with low mitochondrial membrane potential (MMP) and a higher percentage of spermatozoa with SDF and phosphatidylserine externalization, an early marker of apoptosis [28]. The evaluation of MMP is an important marker of sperm mitochondrial function that positively correlates with sperm motility. Mitochondria have a pivotal role in sperm motility and fertilization since they provide energy through glycolysis and oxidative phosphorylation. In spermatozoa, mitochondrial Complex I and Complex III are the major sites for ROS production [36]. As mitochondria are the major source of pro-oxidative agents, the dysfunction of this organelle would have a fundamental role in the oxidative imbalance that affects sperm function [36]. Wang and colleagues identified low MMP and high ROS production in spermatozoa from infertile patients, identifying it as likely being a consequence of this type of mitochondrial dysfunction [37]. We previously reported a relationship between an increased percentage of spermatozoa with low MMP and an increased rate of spermatozoa with abnormal chromatin compactness, suggesting that mitochondrial damage can alter sperm DNA without fragmenting it [38]. According to these results, Kort and colleagues found a significantly higher SDF index in obese compared to normal-weight men [39]. Similarly, in 2009, Chavarro and colleagues reported an increased number of spermatozoa with SDF, which was evaluated by COMET assay, in obese men compared to normal-weight men [13].

**Figure 1:**
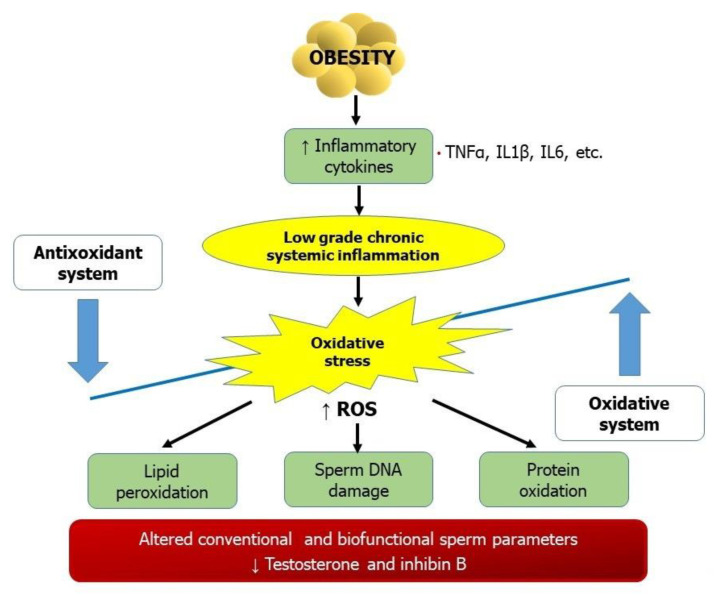
Oxidative stress, obesity, and male infertility.

Among the several mechanisms that are responsible for sperm damage in obese males, the state of systemic inflammation plays a pivotal role (Figure 1). Obesity is a clinical example of systemic oxidative stress. Indeed, obesity is associated with the increased production of pro-inflammatory cytokines, such as interleukin-6 (IL-6) and tumor-necrosis factor α (TNFα), leading to a low chronic inflammatory state [40]. Reactive oxygen species (ROS) are products of normal cellular metabolism and are essential for physiological processes. At physiological levels, ROS stimulates capacitation and the acrosome reaction. In contrast, at high concentrations, they can oxidize, and in turn, damage DNA, proteins, and lipids [41]. Human seminal fluid contains numerous systems with antioxidant activity to counteract the negative effects of ROS. However, an imbalance between oxidant production and antioxidant capacity increases oxidative stress. Spermatozoa are particularly sensitive to the detrimental effects of increased oxidative stress because their plasma membrane is constituted by high levels of polyunsaturated fatty acids (PUFA). The double bonds of the membrane lipids can be oxidized by ROS, and this causes a process called “lipid peroxidation” and, in turn, a reduction of membrane fluidity. Moreover, ROS can directly damage sperm DNA [42]. Spermatozoa are unable to repair DNA due to the lack of the cytoplasmic enzyme systems that are involved in the molecular mechanisms for DNA repair [42]. Lipid peroxidation and mitochondrial superoxide levels measurements are sensible oxidative stress indices. According to recent evidence, 8-hydroxy,2-deoxyguanosine (8-OH-DG) can be used as an index of oxidative stress on DNA, demonstrating high sensitivity and specificity [43].

Obesity is characterized by an increased production of pro-inflammatory cytokines, such as interleukin-6 (IL-6) and tumor-necrosis factor α (TNFα), leading to a low chronic inflammatory state. An imbalance between oxidative and antioxidant system provokes an increased production of reactive oxygen species (ROS). At physiological levels, these are essential for physiological processes; in contrast, at high concentrations, they can oxidize, and, in turn, damage DNA, proteins, and lipids.

## 7. Obesity and Seminal Plasma Proteome

In recent years, interest in proteomics has grown rapidly. The term “proteomics”, which was first coined in 1995, was defined as the large-scale analysis of the protein content of a cell, tissue, or biological fluid [44]. Recently, several studies have focused on the expression pattern of proteins in the seminal plasma of patients with increased oxidative stress, including obesity [45]. The seminal plasma is constituted by secretions originating from the seminal vesicles (65%), prostate (25%), epididymis, and testis (10%) [45]. It has a key role in sperm survival and quality. The proteomic analysis of seminal plasma proteome could provide important biomarkers of oxidative stress in spermatozoa. Indeed, the seminal plasma proteome can be impaired by ROS overproduction [46]. Recently, Herwig and colleagues reported the overexpression of proteins that are involved in oxidative stress response, such as haptoglobin (HP) and the protein S100 calcium-binding protein A9 (S100A9) in the seminal plasma of obese men [47]. Specifically, the S100A9 protein belongs to a group of calcium-binding proteins that have the EF-hand domain and that bind to pro-inflammatory receptors to start the inflammatory cascade. Thus, its overexpression represents an index of the inflammatory state that is induced by oxidative stress. On the contrary, HP is a late positive acute-phase protein of inflammation, and it has antioxidant effects. Moreover, the authors described increased levels of other proteins that are involved in antioxidant activity, specifically ceruloplasmin, clusterin, glutathione peroxidase, mitochondrial glutathione reductase, and adenosine diphosphate (ADP) ribosyl cyclase in the seminal plasma of obese men [47]. Therefore, the overexpression of these proteins probably represents an attempt to maintain the balance between oxidant and antioxidant systems and, in turn, to protect cells from the damage induced by excessive ROS production [48]. Changes in the seminal plasma proteome profile suggest the latter’s role in impaired reproductive function in obese men in cases where increased oxidative stress is observed.

## 8. Adipokines, Obesity, and Male Reproduction

Until the 1980s, adipose tissue was considered a simple inactive store of energy in the form of triglycerides. However, it is now known that adipose tissue is an active endocrine organ that is responsible for the production of several hormones. These include adipokines and immune molecules (cytokines and chemokines) [49]. In physiological conditions, the molecules that are produced by adipose tissue contribute to the maintenance of body homeostasis and can modulate the activity of the immune system [50]. However, in obese people, a dysregulation of the adipokine and cytokine pathways causes the development of chronic low-grade inflammation, leading to the chronic complications of obesity. Hypertrophic adipocytes have pro-inflammatory potential and promote insulin resistance. In obese patients, non-esterified fatty acids (NEFA) that are produced by hypertrophic adipocytes induce local macrophages to secrete high levels of TNFα, which, in turn, stimulates adipocytes to produce more NEFA, pro-inflammatory cytokines (interleukin 1ß (IL1β) and IL6), acute phase proteins, and chemokines [+(C-C motif chemokine ligand-2 (CCL2) or monocyte attractant protein-1 (MCP-1)), which attract more monocytes/macrophages within adipose tissue [51]. Thus, a vicious cycle is established that promotes the inflammation of the adipose tissue first and then a systematic state of low-grade inflammation [49]. Among adipokines, leptin is the most studied in male fertility, whereas fewer data are available for the others.

### 8.1. Leptin

Leptin, a 16-kDa adipokine, was first identified as a product of the adipose tissue in 1994 [52]. It is encoded by the *Obesity* (*Ob*) gene that is expressed in adipocytes, and its circulating levels are positively associated with the percentage of body fat and adipocyte size [53]. Leptin is considered the “satiety hormone”. It binds its receptors in the hypothalamus and promotes the repression of genes encoding neuropeptide Y (NPY) and the induction of genes encoding proopiomelanocortin (POMC) and the amphetamine-regulated transcript (CART). This results in decreased appetite and lower food intake. In addition to its role in the regulation of food intake, leptin has been shown to have important effects on reproduction, acting both as a central and peripheral factor [54]. Leptin-deficient *ob/ob* mice are usually infertile, and treatment with leptin restores fertility [55]. In vitro and in vivo studies have shown that leptin stimulates LH secretion [56]. However, the regulation of leptin on hypothalamic GnRH secretion is not direct because GnRH neurons do not express leptin receptors.

The central role of leptin on reproduction seems to be mediated by kisspeptin. Indeed, kisspeptin neurons are mainly located in the infundibular nucleus and the rostral preoptic area of the hypothalamus and kisspeptin receptor GPR54, an orphan G protein-coupled receptor that is expressed in GnRH neurons. Kisspeptin stimulates GnRH release and, in turn, LH, FSH, and T secretion [57]. Therefore, kisspeptin does not have a direct effect on the pituitary gland and is only able to affect it via GnRH secretion. The leptin receptor has six isoforms and activates different signaling pathways, among which the Janus kinase (JAK) and signal transducer and activator of transcription proteins (STAT3 and STAT5) are the best characterized. However, other leptin-signaling pathways involve the mitogen-activated protein kinase (MAPK)/extracellular signal-regulated kinase (ERK), phosphoinositol 3 kinase (PI3K), mammalian target of rapamycin (mTOR), and/or nitric oxide [58].

**Figure 2:**
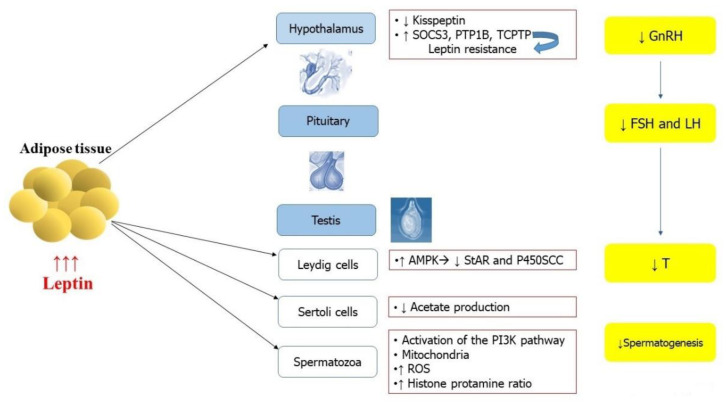
Effects of hyperleptinemia on male reproductive system. Abbreviations: GnRH: gonadotropin releasing hormone; LH: luteinizing hormone; FSH: follicle-stimulating hormone; SHBG: sex hormone-binding globulin; E_2_: 17β-estradiol. T: testosterone; AMPK: 5′ adenosine monophosphate-activated protein kinase is an enzyme; StAR: steroidogenic acute regulatory protein; P450SCC: cytochrome P450 family 11 subfamily A member 1; PI3K:phosphatidylInositol 3-Kinase.

Although leptin has an essential role in the regulation of reproduction, elevated levels of this adipokine impact negatively male fertility (Figure 2). In obesity, where excessive amounts of leptin are produced by adipocytes, the hypothalamic–pituitary axis becomes resistant to leptin signaling, presumably by over-activating negative feedback regulators, including the suppressor of cytokine signaling 3 (SOCS3), protein tyrosine phosphatase 1B (PTP1B), and T cell protein tyrosine phosphatase (TCPTP). The expression of these negative regulators is elevated in the hypothalamus of obese animals [59].

Animal studies have shown that a high-fat diet and central leptin resistance decrease kisspeptin expression both in the rostral periventricular region of the third ventricle and the arcuate nucleus [58]. Therefore, decreased kisspeptin secretion plays a pivotal role in obesity-related central hypogonadism by inhibiting GnRH neurons and, in turn, decreasing gonadotropins and T secretion.

A positive correlation was described between serum leptin levels, BMI, and the alteration of sperm parameters in both animals and humans. Leptin is also present in the testis and in the seminiferous tubules in particular [60].

Animal studies have shown that the intraperitoneal administration of leptin, in doses ranging from 5 to 60 µg kg^−1^, for six weeks to normal-weight Sprague Dawley rats, caused a significant decrease in sperm count and the percentage of spermatozoa with normal morphology compared to normal-weight untreated rats [54,61,62,63]. In addition, it was reported that the administration of leptin results in increased levels of ROS [62], 8-OH-DG, and SDF in rats [62,63]. In humans, a case–control study showed that obese men have high levels of leptin, low sperm concentrations, and increased SDF compared to normal-weight men [64]. Therefore, leptin also seems to act directly at the testicular level, increasing the production of ROS [54]. Moreover, although the previous findings are controversial, leptin receptors also seem to be present in spermatozoa, specifically on the tail [65]. Previous studies suggest a dual role of leptin on sperm parameters according to its concentration in the seminal plasma. Elfassy and colleagues hypothesized that leptin has a beneficial role on semen at physiological concentrations but a negative effect at high concentrations, corresponding to those found in overweight/obese men [66]. More specifically, leptin binds its receptors in the male reproductive system and activates the PI3K pathway in the testis, increasing oxidative stress and also disrupting the histone-to-protamine transition. An abnormal protaminization exposes the sperm DNA to attack by free radicals, resulting in increased SDF and apoptosis [54].

In men, leptin levels correlate inversely with T levels. In vitro studies of rat LCs showed that leptin at concentrations similar to those reached in obesity inhibited testosterone secretion [67,68]. Specifically, hyperleptinemia may up-regulate the AMPK pathway, and this up-regulation inhibits the activation of the steroidogenic acute regulatory protein (StAR) and the rate-limiting steroidogenic enzyme cytochrome P450 family 11 subfamily A member 1 (P450SCC) levels in LCs. Therefore, the high leptin levels that are found in obese individuals down-regulate STAT transcriptional activity, leading to lower expression levels of the cAMP-dependent steroidogenic genes that are involved in T production.

Moreover, increased levels of leptin also seem to impair the nutritional function of SCs the spermatogenesis “nurse cells”. SCs take glucose from circulation and produce the metabolites that are needed for germ cell development. In cultured human SCs, the physiological concentration of leptin (5 ng/mL) upregulates glucose transporter 2 proteins (GLUT-2) levels, whereas obesity levels of leptin (25–50 ng/mL) decreased acetate production from glucose in a dose dependent-manner [69]. These results suggest that hyperleptinemia can negatively impact LC testosterone production and the metabolic support of SCs towards spermatogenesis [58].

In addition, leptin also modulates mitochondrial activity, and it is associated with mitochondrial dysfunction in different cells, including cardiomyocytes. Mitochondria are the main source of superoxide anions in cells. Therefore, alterations in mitochondria function due to high leptin levels could result in an increase in oxidative stress and, in turn, can contribute to the leptin-induced adverse effects on the spermatozoa [70].

Leptin has an essential role in the regulation of reproduction; however, in obesity, increased levels of this adipokine are produced by adipocytes. These excessive amounts may impair the hypothalamic–pituitary–gonadal axis through different mechanisms and at different levels. In the hypothalamus, the expression of negative regulators such as the suppressors of cytokine signaling 3 (SOCS3), protein tyrosine phosphatase 1B (PTP1B), and T cell protein tyrosine phosphatase (TCPTP) increase in obesity, and, in turn, the hypothalamic–pituitary axis becomes resistant to leptin signaling. Moreover, hyperleptinemia reduces kisspeptin expression and, as a consequence, inhibits GnRH neurons. The inhibition of the GnRH neurons provokes a reduced release of GnRH and, in turn, of LH, FSH, and testosterone. Elevated levels of leptin may also directly affect testes function at different levels (Leydig cells, Sertoli cells, and spermatozoa). 

### 8.2. Adiponectin

Adiponectin is a 28 kDa protein with a similar structure to TNFα, collagen VIII and IV, and complement factor C1q [50]. Unlike the majority of adipokines, it exerts an anti-inflammatory effect and negatively correlates with body fat mass [50].

Adiponectin and its receptors (AdipoR1 and AdipoR2) are expressed in the pituitary gland, suggesting a central role for this adipokine [49]. In addition, animal studies also suggest a direct effect of adiponectin on the testis, where it regulates steroidogenesis and spermatogenesis [49]. Accordingly, adiponectin receptors are present in LCs, SCs, and germ cells in rats [66]. Adiponectin has been found in circulation in various molecular isoforms: low (LMW), medium (MMW), and high molecular weight (HMW). The HMW form of adiponectin is the predominant circulating one (>80%) [66]. The concentrations of adiponectin are 66-fold lower in human seminal plasma compared to serum [71]. This may be due to the blood–testis barrier that prevents the passage of huge molecules. It was reported that seminal adiponectin concentrations are lower in overweight or obese patients compared to normal-weight patients [71]. Adiponectin levels in the seminal plasma have been shown to positively correlate with sperm concentration, total sperm count, and spermatozoa with normal morphology [66,71]. As previously discussed, pro-inflammatory cytokines, such as TNF-α and IL-1β, can inhibit LC function, steroidogenesis, and spermatogenesis. Adiponectin may protect LCs from the negative effect of inflammatory cytokines [72]. Specifically, adiponectin inhibits the formation and translocation to the nucleus of the NF–Kb complex through the promotion of AMP-activated protein kinase phosphorylation [72]. Therefore, adiponectin is associated with a down-regulation of prof-inflammatory genes, a reduced production of pro-inflammatory cytokines, such as TNFα, IL-6, and an increased release of anti-inflammatory cytokines (IL-10) [73]. Moreover, unlike many adipokines, adiponectin has an insulin-sensitizing effect [73].

### 8.3. Resistin

Resistin is a 12 kDa polypeptide that belongs to a family of cysteine-rich proteins [49]. It is involved in the development of insulin resistance, and its levels positively correlate with inflammatory markers [50]. The presence of resistin was described in the testis and, specifically, in both the LCs and seminiferous tubules of rats, but it was not reported in humans [66]. A very low number of studies have evaluated the presence of resistin in the seminal plasma [66]. One of these studies described a negative correlation between the concentrations of seminal resistin levels and sperm motility and vitality [74]. Moreover, the authors also reported higher levels of resistin in patients with leukocytospermia and a positive correlation with other inflammation markers in semen, such as IL-6 and elastase [74]. Therefore, seminal resistin could have an important role in the inflammation of the male reproductive system [49]. However, other studies did not find any significant correlation between sperm parameters and resistin [71]. Furthermore, resistin could also regulate reproduction through a central mechanism [49]. Indeed, resistin was described in the hypothalamus and pituitary gland of both humans and animals [75].

### 8.4. Visfatin

Visfatin is a 52 kDa protein, and it is also known as nicotinamide phosphorybosyltrasferase (NAMPT) [49]. In obesity, its role is still controversial [50]. Some studies suggest that increased visfatin levels could exert a protective role [76]. Conversely, other studies showed an adverse effect of this adipokine on insulin resistance [77]. A meta-analysis reported that visfatin levels are increased in patients with obesity/overweight, diabetes mellitus, metabolic syndrome, and cardiovascular disease [78]. Visfatin has been found in LCs, spermatocytes, and spermatozoa [66]. Interestingly, its levels are higher in seminal plasma than they are in blood. This suggests that testicular cells produce NAMPT [71]. In cultured LCs, visfatin has been shown to increase T secretion [79].

### 8.5. Chemerin

More recently, chemerin has been identified among adipokines. It consists of 163 amino acids, and it modulates insulin action [80]. Serum chemerin concentrations are significantly higher in overweight and obese patients compared to those with normal weight, and its levels are positively associated with BMI [71]. Moreover, it was also described to have a significant negative association between chemerin and serum T levels [71]. The exact mechanisms through which chemerin can modulate male reproduction are still unclear. In humans and rats, chemerin and its receptors are present in the testis, particularly in LCs [81,82]. In vitro studies have shown an inhibitory effect of chemerin on spermatogenesis [81,82]. Indeed, chemerin inhibits the secretion of T in response to human chorionic gonadotropin (hCG) in primary LC cultures. Moreover, a human study reported a negative correlation between the chemerin levels and sperm motility in seminal plasma but a positive correlation with sperm concentration [71].

## 9. Sirtuins, Obesity, and Male Reproduction

Sirtuins (SIRTs) are highly conserved nicotinamide adenine dinucleotide (NAD^+^)-dependent deacetylases that are involved in gene regulation, metabolism, aging, and cancer [83]. The SIRT family is composed of seven members (SIRT1-SIRT7) that have different subcellular localizations, particularly SIRT1, SIRT6, and SIRT7 are mostly found in the nucleus, SIRT2 is localized in the cytosol, whereas SIRT3, SIRT4, and SIRT5 are localized in the mitochondria [84]. The emerging role of SIRTs as key metabolic sensors for body homeostasis has resulted in a growing interest in these molecules in the scientific literature [83]. Indeed, SIRTs regulate metabolic homeostasis in different tissues through the activation of several pathways and enzymes, including peroxisome proliferator-activated receptor-gamma coactivator (PGC)-1α, peroxisome proliferator-activated receptor gamma (PPAR-γ), adenosine monophosphate-activated protein kinase (AMPK), forkhead box transcription factor O1 (FOXO1), and liver x receptor (LXR) [85]. SIRTs are also expressed in adipose tissue and affect adipogenesis by transcriptionally regulating PPAR-γ, a master regulator of adipocyte differentiation.

SIRT1, the most studied member of the SIRT family, regulates cellular metabolism, oxidative stress, and mitochondrial biogenesis [86]. Its levels are significantly decreased in the fatty tissue of humans and mice with obesity [87]. Based on murine cell culture models, it has been shown that the repression of PPAR-γ by the overexpression of SIRT1 or SIRT2 blocks adipocyte differentiation, whereas reduced SIRT1 or SIRT2 levels enhance it [88]. Moreover, when incorrectly stimulated, SIRTs appear to be critical for the inflammatory and metabolic response to the hypoxic visceral adipose tissue microenvironment that is typical of the obese state [88].

In addition to their role as metabolic sensors, SIRTs seem to have an important function in male fertility. They are highly expressed in mammalian testicular tissue [89]. Specifically, SIRT1 was identified in the nuclei of spermatogonia, spermatocytes, and round spermatids, suggesting a role of SIRT1 in spermatogenesis [90]. SIRTs control testicular function by regulating different mechanisms that are fundamental for spermatogenesis, including glycolysis, the oxidation of fatty acids, chromatin remodeling, and oxidative stress. Specifically, SIRT1 regulates glycolysis via the inhibition of the hypoxia-inducible factor-1 (HIF-1) transcription factor. The latter controls the expression of genes that are crucial for glycolysis [91], including glucose transporter 1 and lactate dehydrogenase A. SIRT1 can deacetylate and inhibit HIF-1 and consequently inhibits the expression of the genes that are important for glycolysis [91]. SIRT3 seems also to be involved in the regulation of glycolysis even though it is a mitochondrial molecule [83,92]. The lactate that is produced by SCs is one of the major substrates for germ cell metabolism; thus, the regulation of glucose metabolism by SIRTs is fundamental for spermatogenesis [92].

SIRTs also appear to be involved in lipid metabolism, which plays an essential role in steroidogenesis to ensure an adequate energy supply for SCs. SIRT3 is important for the deacetylation of the main enzymes that are involved in β-oxidation, such as long-chain acylCoA dehydrogenase (LCAD). Thus, reduced levels of SIRT3 are associated with reduced fatty acid oxidation [93].

SIRTs are also crucial for mitochondrial sperm function, which is strictly correlated with sperm motility [94]. As previously mentioned, both in vitro and in vivo studies have reported that SIRT1 is a regulator of PCG-1α, a transcriptional coactivator that increases the activity of the enzymes that are involved in mitochondrial processes [95]. In mice, the absence of SIRT-1 was associated with an arrest of spermatogenesis due to dysfunctional mitochondria and declined testicular bioenergetic capacity [96]. SIRT3 modulates mitochondrial energy homeostasis by regulating adenosine triphosphate (ATP) generation from oxidative phosphorylation [83].

Furthermore, SIRT1 seems to protect cells from oxidative stress damage. Previous studies have reported that the activation of SIRT1 decreases malondialdehyde (MDA) levels and increases superoxide dismutases and glutathione peroxidase levels [97], contributing to the maintenance of a balance between oxidative and antioxidant systems. In contrast, reduced levels of SIRT1 and SIRT3 lead to a defective function in the electron transport chain, increasing the production of ROS.

Most of the evidence on the role of SIRTs in male reproduction has been obtained by knockout (KO) mice with altered SIRT1 activity. This experimental model has clearly shown the role of SIRTs in spermatogenesis. SIRT1 KO mice have a decreased sperm count and normal sperm morphology and an increased SDF [98]. The testes of SIRT1 KO mice are characterized by an important reduction of spermatids that can potentially result in their total absence [90,99]. It has been hypothesized that SIRT1 deficiency may alter spermatogenesis through different mechanisms, including the up-regulation of p53 activity and ensuing testicular apoptosis or by an increase in oxidative stress [100].

In addition, SIRTs are highly expressed in the hypothalamus [101], particularly in GnRH neurons [102]. They may disrupt the HG axis function. Indeed, SIRT1 KO mice demonstrate the reduced secretion of hypothalamic GnRH and consequently a significant decrease in LH and FSH serum levels [99]. The authors also observed an important down-regulation of steroidogenic acute regulatory (StAR), the enzyme that is responsible for the rate-limiting step of steroidogenesis, in SIRT1 KO mice. These mechanisms result in a reduction of T secretion.

Previous studies have evaluated the possibility of interfering with SIRT activity through pharmacological and non-pharmacological interventions. As for the latter, caloric restriction correlates with an increase in SIRT1 levels in muscle tissue [103]. On the other hand, among the compounds that are able to regulate the activity of SIRTs, one of the first to be studied was resveratrol (3,5,4′-trihydroxystilbene), a natural polyphenol that is present in grapes, berries, and red wine [34] and demonstrate a strong antioxidant capacity. The administration of resveratrol seems to improve the metabolic profile of obese and/or insulin-resistant patients through various mechanisms, including the enhancement of SIRT1 activity [104]. Interestingly, resveratrol also improves sperm motility and mitochondrial activity and counteracts sperm damage induced by oxidative stress [105]. Furthermore, high-fat-fed mice treated with resveratrol showed a recovery of serum T levels due to an increase in the activity of the StAR enzyme [106]. The regulation of SIRT activity may bring about several health benefits, including the prevention and treatment of obesity [86].

## 10. Obesity and Epigenetic Modifications

There are increasing data that paternal obesity may negatively impact the reproductive and metabolic health of offspring. Epidemiological studies have shown that children born from obese fathers are more likely to be obese [107]. Epigenetics has offered new explanations on the mechanisms through which environmental factors can modulate the expression of genes [108]. Epigenetics is defined as the processes that alter gene activity without nucleotide sequence modification but that include chromatin structure changes [109]. The most relevant epigenetic mechanisms that are involved in gene activity regulation are DNA methylation, histone modifications, and non-coding RNAs. Many studies on epigenetic effects of obesity are conducted in mothers, and the epigenetic effects of obesity are more rarely examined. Among these mechanisms, DNA methylation is the most widely studied [110]. It involves the covalent addition of a methyl group (-CH3) onto the position five of cytosine, resulting in 5-methylcytosine.

DNA methylation is essential for spermatogenesis. Sperm methylation is necessary to inactive the X chromosome during meiosis and for the establishment of paternally imprinted genes in the spermatozoa [111]. The abnormal methylation of sperm imprinted genes seems to be involved in infertility and pregnancy loss [112]. Interestingly, significantly altered DNA methylation has been observed in the spermatozoa of overweight and obese men compared to normal-weight men in the regulatory region of many imprinted genes [113]. Among the genes that undergo epigenetic modification in obese males, the following are included: maternally expressed gene 3 (*MEG3*), necdin (*NDN*), small nuclear ribonucleoprotein polypeptide N (*SNRPN*), and epsilon sarcoglycan (*SGCE*)/paternally expressed gene 10 (*PEG10*), which are involved in fetal development and tumor growth [114]. Donkin and colleagues evaluated an epigenetic mapping in the spermatozoa of obese and normal-weight men [115] and found significant different methylation between the two groups. The hypomethylation of imprinted genes was associated with higher levels of SDF and decreased pregnancy rates [116,117]. Moreover, babies born to obese fathers have altered DNA methylation in the regulatory regions of several imprinted genes [114]. Previous studies have suggested that obesity-related epigenetic changes in spermatozoa may be reversed by weight loss. This decreases the chances of an adverse impact on the offspring’s health [118]. Research on epigenetic biomarkers is required to better understand the pathophysiology of obesity and its clinical manifestations in future generations.

## 11. Conclusions

**Figure 3:**
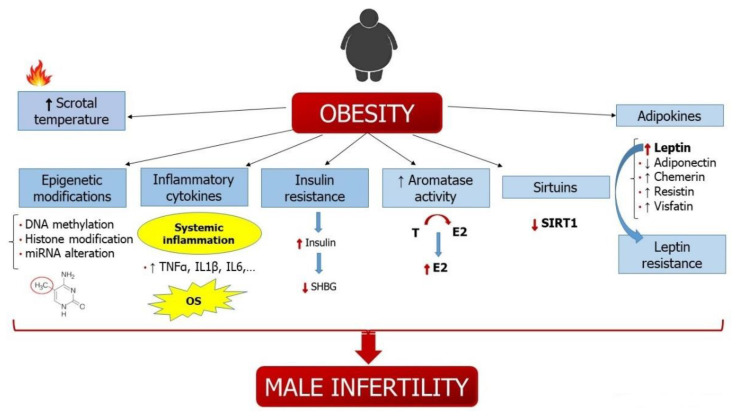
Main molecular mechanisms involved in the effects of obesity on male infertility. Abbreviations: GnRH: gonadotropin releasing hormone; LH: luteinizing hormone; FSH: follicle-stimulating hormone; SHBG: sex hormone-binding globulin; E_2_: 17β-estradiol. T: testosterone.

An unhealthy attitude towards food and a sedentary lifestyle has greatly increased the prevalence of obesity in Western countries. In parallel, a progressive decline in sperm quality and male fertility has been described in recent decades [17,119]. Several studies have amply demonstrated the close association between obesity and reproductive dysfunction. Although the relationship between female fertility and obesity has been more investigated, the attention to obesity-induced male infertility has gained increasing interest in recent decades and is gaining more and more evidence. The mechanisms by which obesity impairs male reproductive function are many and complex (Figure 3), but the precise mechanisms remain to be fully elucidated. Obesity disrupts the hormonal milieu, alters conventional and biofunctional sperm parameters, induces systemic inflammation, increases oxidative stress, and promotes epigenetic modifications that can also be transmitted to offspring.

In the intriguing and complex web of the molecular mechanisms that are behind obesity-induced male infertility, new molecules are appearing on the horizon, such as adipokines and SIRTs. Adipokines, which are secreted by adipose tissue, can deeply influence male reproduction. Among these, leptin is the most studied [70]. Although leptin is fundamental for normal reproductive function, an excess of this adipokine, as observed in obese patients, can severely impair the male reproductive system [70]. Leptin and other adipokines represent an important link between the adipose tissue and the reproductive system at different levels (both central and gonads) in men [49]. Evidence also suggests a clear role of SIRTs on obesity and male fertility through different mechanisms. Therefore, the activation or inhibition of SIRT activity could have positive effects on obesity and male reproduction, as shown by previous studies using resveratrol, which modulates SIRT activity [104,105].

Understanding the molecular mechanisms behind the association between obesity and male fertility could increase our capacity to identify novel targets for the prevention and the treatment of obesity and obesity-related diseases, including those affecting the male reproductive system.

The mechanisms by which obesity impairs male reproductive function are many and complex. The excessive visceral adipose alters the hormonal milieu in obese male patients, decreasing the levels of SHBG, total and free T, and inhibin B and increasing the conversion of T into E_2_ due to greater aromatase activity. The excess visceral fat also causes insulin resistance as well as an increase in insulin levels, which decrease SHBG production in the liver, leading to higher levels of E2. The excess of E2 inhibits the HPG axis and, in turn, results in a reduction of T production. The fat accumulation in the suprapubic region increases scrotal temperature in obese men, resulting in impaired sperm parameters and increased oxidative stress. Obesity also promotes epigenetic modifications trough different mechanisms, including DNA methylation, histone modification, and miRNA alteration, which can also be transmitted to offspring. Adipose tissue also secretes pro-inflammatory cytokines, which induce low-grade systemic inflammation. The production of adipokines is altered in obese patients. The excess leptin and the consequent leptin resistance impairs male fertility at both the central and peripheral levels. In fact, Leptin can modulate GnRH production, mainly via Kisspeptin, and can also directly act on spermatogenesis. Moreover, sirtuins can play an important role in obesity-induced male infertility. The levels are significantly decreased in the fatty tissue of obese patients. Sirtuins control testicular function by regulating different mechanisms that are fundamental for spermatogenesis. Decreased levels of SIRT1 are associated with a reduction in GnRH, LH, and FSH production and with an alteration of spermatogenesis. All together, these mechanisms lead to abnormal conventional and biofunctional sperm parameters, an increase of sperm DNA damage, and an alteration of sex hormones in obese patients.
